# Long COVID and cardiovascular disease: a prospective cohort study

**DOI:** 10.1136/openhrt-2024-002662

**Published:** 2024-05-27

**Authors:** Claire Alexandra Lawson, Alastair James Moss, Jayanth Ranjit Arnold, Catherine Bagot, Amitava Banerjee, Colin Berry, John Greenwood, Alun D Hughes, Kamlesh Khunti, Nicholas L Mills, Stefan Neubauer, Betty Raman, Naveed Sattar, Olivia C Leavy, Matthew Richardson, Omer Elneima, Hamish JC McAuley, Aarti Shikotra, Amisha Singapuri, Marco Sereno, Ruth Saunders, Victoria Harris, Linzy Houchen-Wolloff, Neil J Greening, Ewen Harrison, Annemarie B Docherty, Nazir I Lone, Jennifer Kathleen Quint, James Chalmers, Ling-Pei Ho, Alex Horsley, Michael Marks, Krisnah Poinasamy, Rachael Evans, Louise V Wain, Chris Brightling, Gerry P McCann

**Affiliations:** 1 Department of Cardiovascular Sciences, University of Leicester, Leicester, UK; 2 Department of Haemostasis and Thrombosis, Glasgow Royal Infirmary, Glasgow, Glasgow, UK; 3 Farr Institute of Health Informatics Research, University College London, London, UK; 4 BHF Glasgow Cardiovascular Research Centre, University of Glasgow, Glasgow, UK; 5 Cardiology, Golden Jubilee National Hospital, Clydebank, UK; 6 Cardiology, Leeds Teaching Hospitals NHS Trust, Leeds, UK; 7 Biomedical Imaging Sciences, University of Leeds, Leeds, UK; 8 MRC Unit for Lifelong Health and Ageing, University College London, London, UK; 9 Leicester Real World Evidence Unit, Leicester Diabetes Centre, University of Leicester, Leicester, UK; 10 BHF Centre for Cardiovascular Sciences, University of Edinburgh, Edinburgh, Edinburgh, UK; 11 Radcliffe Department of Medicine, University of Oxford, Oxford, UK; 12 Institute of Cardiovascular and Medical Sciences, British Heart Foundation Glasgow Cardiovascular Research Centre, University of Glasgow, Glasgow, UK; 13 Department of Population Health Sciences, University of Leicester, Leicester, UK; 14 University of Leicester, Leicester, UK; 15 University of Edinburgh, Edinburgh, UK; 16 NHLI, Imperial College London, London, UK; 17 University of Dundee, Dundee, Dundee, UK; 18 University of Oxford, Oxford, UK; 19 University of Manchester, Manchester, UK; 20 Department of Clinical Research, London School of Hygiene and Tropical Medicine, London, UK; 21 Asthma and Lung UK, London, UK; 22 The Institute for Lung Health, University of Leicester, Leicester, UK; 23 Department of Cardiovascular Sciences, Glenfield Hospital, Leicester, UK

**Keywords:** CARDIAC REHABILITATION, COVID-19, RISK FACTORS

## Abstract

**Background:**

Pre-existing cardiovascular disease (CVD) or cardiovascular risk factors have been associated with an increased risk of complications following hospitalisation with COVID-19, but their impact on the rate of recovery following discharge is not known.

**Objectives:**

To determine whether the rate of patient-perceived recovery following hospitalisation with COVID-19 was affected by the presence of CVD or cardiovascular risk factors.

**Methods:**

In a multicentre prospective cohort study, patients were recruited following discharge from the hospital with COVID-19 undertaking two comprehensive assessments at 5 months and 12 months. Patients were stratified by the presence of either CVD or cardiovascular risk factors prior to hospitalisation with COVID-19 and compared with controls with neither. Full recovery was determined by the response to a patient-perceived evaluation of full recovery from COVID-19 in the context of physical, physiological and cognitive determinants of health.

**Results:**

From a total population of 2545 patients (38.8% women), 472 (18.5%) and 1355 (53.2%) had CVD or cardiovascular risk factors, respectively. Compared with controls (n=718), patients with CVD and cardiovascular risk factors were older and more likely to have had severe COVID-19. Full recovery was significantly lower at 12 months in patients with CVD (adjusted OR (aOR) 0.62, 95% CI 0.43 to 0.89) and cardiovascular risk factors (aOR 0.66, 95% CI 0.50 to 0.86).

**Conclusion:**

Patients with CVD or cardiovascular risk factors had a delayed recovery at 12 months following hospitalisation with COVID-19. Targeted interventions to reduce the impact of COVID-19 in patients with cardiovascular disease remain an unmet need.

**Trail registration number:**

ISRCTN10980107.

**WHAT IS ALREADY KNOWN ON THIS TOPIC**

- In unselected populations fewer than one in three patients hospitalised with SARS-CoV-2 report complete recovery after 1 year. Whether pre-existing cardiovascular disease or cardiovascular risk factors limit recovery following SARS-CoV-2 hospitalisation remains unknown.

**WHAT THIS STUDY ADDS**

- In a large prospective cohort study, pre-existing cardiovascular disease and cardiovascular risk factors were associated with lower rates of complete recovery up to 1 year after SARS-CoV-2 hospitalisation.

**HOW THIS STUDY MIGHT AFFECT RESEARCH, PRACTICE OR POLICY**

- Patients with established cardiovascular disease are at high risk of deteriorating physical and mental health following SARS-CoV-2 hospitalisation. Targeted interventions to reduce the impact of respiratory infections in these individuals should be a priority for public health policy.

## Introduction

Hospitalisation with severe acute respiratory syndrome-coronavirus-2 (SARS-CoV-2) infection was associated with high rates of morbidity, particularly in those with underlying chronic conditions such as cardiovascular disease (CVD) before the availability of vaccination programmes.^1^ In severe cases of SARS-CoV-2 infection, complete resolution of symptoms is rarely achieved during the acute hospital admission and a period of convalescence is required to monitor symptoms outside of the hospital setting. Persisting functional limitations align with a group of symptoms that overlap with those of CVD, including exercise intolerance, breathlessness, chest pain, palpitations and fatigue, thereby posing a diagnostic and management challenge for physicians and significant anxiety to patients.^2^ Establishing whether these symptoms are attributable to SARS-CoV-2 infection or due to the development of alternative pathology is a key priority for patients and healthcare services at this stage in the coronavirus disease 19 (COVID-19) pandemic, thereby placing a considerable strain on finite healthcare resources.^3 4^

To address the uncertainty encountered by patients discharged from hospitals with COVID-19, multidisciplinary clinics to monitor the post-acute sequelae of COVID-19, termed long COVID, were established within the UK National Health Service (NHS) in 2020. Long COVID, as defined by the WHO, is a debilitating post-COVID-19 condition with a cluster of signs and symptoms that impact on everyday functioning, develop during or after an initial SARS-CoV-2 infection and continue for more than 12 weeks which cannot be explained by an alternative diagnosis. We have previously reported that the rate of patient-perceived complete recovery was 29% in those hospitalised with COVID-19 at 5 months after discharge.^5^ However, there is considerable variation in the recovery profile, with concomitant mental and physical comorbidities reducing recovery to less than 10% in the most severe cases.^5^ Retrospective analyses from electronic health records have raised doubt as to whether established CVD is a risk factor for long COVID.^6 7^ However, metrics recorded in electronic health records have limitations and more detailed analyses using patient-reported outcomes are warranted. Our primary hypothesis was that the presence of pre-COVID-19 CVD was associated with a lower likelihood of complete recovery at 5 and 12 months compared with those without. In a prospective, national observational cohort study of patients with long COVID, our aim was to determine whether the rate of full recovery was reduced in patients with pre-COVID-19 CVD using patient-reported outcomes which measured physical, physiological and cognitive recovery at 5 and 12 months following COVID-19 hospitalisation.

## Methods

### Study design and participants

The post-hospitalisation COVID-19 study was a prospective cohort study that recruited patients aged 18 years or older following discharge from 1 of 83 NHS hospitals between 1 February 2020 and 31 March 2021 across England, Northern Ireland, Scotland and Wales with admission for confirmed or clinician diagnosed COVID-19.^5 8^ Patients with a confirmed diagnosis of a pathogen other than SARS-CoV-2 and those who presented to the emergency department with COVID-19 but were not admitted to the hospital were excluded. This study included the collection of routine clinical data with linkage to health and social care records (Tier 1) and enhanced clinical data collection and biosampling (Tier 2). For the purposes of this analysis, the study population was restricted to patients who consented to attend up to two follow-up research visits within 1 year of discharge (Tier 2). Participants were stratified into three groups: (1) those with established CVD, which included a documented clinical diagnosis of heart failure, pacemaker or internal cardioverter defibrillator, coronary artery disease, myocardial infarction, peripheral vascular disease, valvular heart disease, atrial fibrillation, stroke, congenital heart disease or other cardiac condition; (2) those at high risk of developing CVD with cardiovascular risk factors including one or more of: treated hypertension, treated hypercholesterolaemia, diabetes mellitus, chronic kidney disease (estimated glomerular filtration rate <60 mL/min/1.73 m^2^), obesity (body mass index >30 kg/m^2^) and a current smoker; (3) controls with neither CVD or risk factors.

The study was approved by the UK national research ethics service (920/YH/0225) and was prospectively registered (ISCRTN10980107). It was performed in accordance with the Declaration of Helsinki. All patients provided written informed consent before any study procedures.

### Patient and public involvement

Patient and public involvement (PPI) have been integral to the PHOSP-COVID study and consortium since its conception. The PHOSP PPI group is co-chaired by NOCRI (Kate Holmes) and BLF/Asthma UK (Krisnah Poinasamy) with the representation of over 10 relevant charities. Members of the ‘Long-COVID Facebook support group’ are closely involved and a Leicester BRC PPI group consisting of people with lived experience of a hospital admission for COVID-19. Patients and the public are embedded within the PHOSP infrastructure including our working groups, core management group and executive and steering groups. Patients were involved in the development of the clinical research study including the overarching aims, choice of outcomes, consent processes and the structure of the study visits. Patients review all patient facing material. We have recently completed a joint patient and clinician research priority questions exercise hosted by advisors from the James Lind Alliance to ensure co-ownership of the direction of PHOSP-COVID research. The data presented in this manuscript starts to answer the co-identified top 10 priorities.^3^

### Study procedures

Study procedures were performed as described previously.^5 8^ Baseline characteristics were measured during the hospital admission with acute COVID-19 including results of a PCR test for SARS-CoV-2. Ethnicity was defined as white, South Asian, black and mixed/other. COVID-19 severity was measured using the WHO Clinical Progression Scale and was determined by the highest level of organ support during the hospital admission.^9^ WHO classes were categorised into hospitalised moderate disease (Classes 4 or 5) including those who required no oxygen therapy or oxygen administrated by mask or nasal prongs and hospitalised severe disease (Classes 6–9) including those who required non-invasive ventilation, high flow nasal oxygenation, mechanical ventilation or extracorporeal membrane oxygenation.^9^

### Outcome measures

The primary outcome was the assessment of full recovery from COVID-19 at 5 and 12 months using patient-perceived responses to the question ‘Do you feel fully recovered?’ which were collected as ‘Yes’, ‘No’ or ‘Unsure’. Full recovery was defined as a ‘Yes’ response to this question and all other responses were classified as incomplete recovery. Secondary outcomes included patient-reported outcome measures which were collected using validated questionnaires for physical recovery (EuroQol 5D-5L Questionnaire, Dyspnoea-12 score, Functional Assessment of Chronic Illness Therapy-Fatigue, General Practice Physical Activity Questionnaire), cognitive recovery (Generalised Anxiety Disorder Assessment (GAD)-7, Patient Health Questionnaire (PHQ)-9, Montreal Cognitive Score) and frailty (Rockwood Frailty Score, Nottingham Activities of Daily Living Score). Additional study-specific questionnaires were completed which detailed participants’ general recovery and symptoms. Physiological measures were undertaken during the study visit including the incremental shuttle walk test, handgrip strength and the short physical performance battery. Circulating biomarkers were measured at each study visit and included C reactive protein, HbA1c and brain natriuretic peptide (BNP) or N-terminal-BNP (or both).

### Statistical analysis

Continuous data were expressed as mean (SD), median (IQR) or numbers (%) as appropriate. Patient characteristics were described by groups and missing data was reported. Characteristics between groups were compared using two sample t-test, analysis of variance, Mann-Whitney or χ^2^ testing as appropriate. For the primary analysis, the OR of full recovery was determined in patients with CVD and cardiovascular risk factors separately using the control group as the referent with and without adjustment for age, sex, ethnicity and COVID-19 severity (WHO Clinical Progression Scale).^9^ Logistic models were fitted, comparing patients with CVD and cardiovascular risk factors to controls to estimate ORs and absolute differences with 95% CIs, respectively. Then for all outcomes, linear models (with 100 bootstrap samples) for continuous outcomes and logistic models for binary outcomes were fitted and used to estimate mean values or absolute percentages, respectively (with 95% CIs). For skewed continuous outcomes, quantile regression was used to estimate median values. All estimates were predicted at the mean age and standardised for the remaining covariates (sex, ethnicity and COVID-19 severity). As the covariates used for adjustment had minimal missing data and were unlikely to be missing at random when conditioned on their observed values, we used complete case analysis to reduce any bias away from the null.^10^ A further prespecified exploratory analysis was performed with adjustment for age, sex, ethnicity, COVID-19 severity and frailty score. A significance level of a p value<0.05 and Stata-BE V.17 was used for the analyses.

## Results

### Baseline characteristics

**Figure 1:**
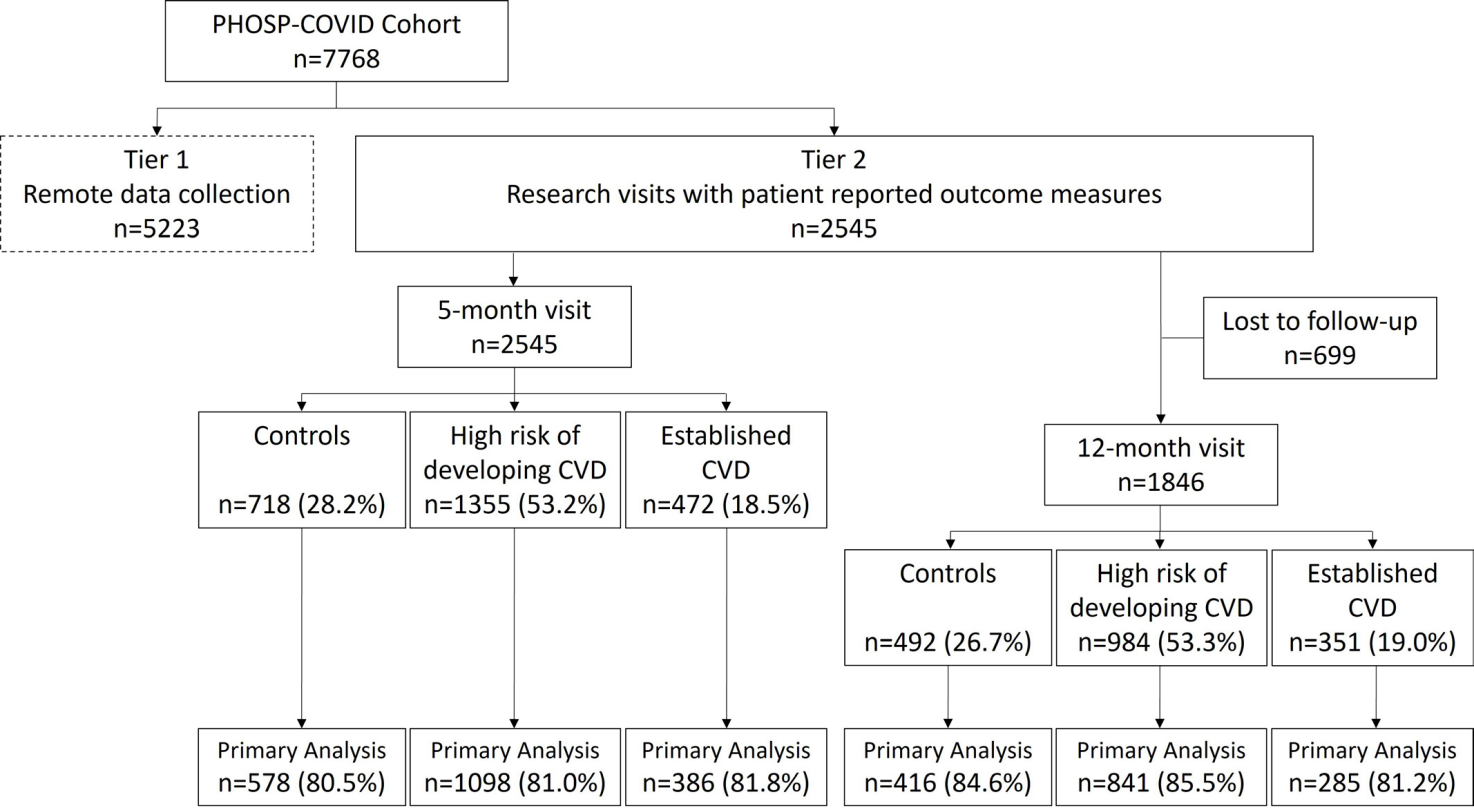
Flow diagram. CVD, cardiovascular disease.

The study population comprised 2545 participants who were discharged from a UK hospital with a diagnosis of COVID-19 between February 2020 and March 2021 and underwent up to two study visits over 12 months (table 1, figure 1). The median time from symptom onset to hospital admission was 8 days (IQR 6–11) with a median hospital length of stay of 8 days (IQR 4–16). Overall, 2164/2545 (93.0%) had a positive PCR test to confirm SARS-CoV-2 infection during the hospital admission, with 1021/2545 (40.1%) classified as having hospitalised severe COVID-19 pneumonitis (WHO Classes 6–9). Of those with hospitalised severe disease, 437/1021 (42.8%) underwent invasive mechanical ventilation during their period of hospital treatment.

**Table 1:** Patient characteristics at time of hospitalisation with COVID-19 stratified by disease/risk group

	Total	Control	High risk of developing CVD	Established CVD	P value	Missing values
	n=2545	n=718	n=1355	n=472		
Age at admission (years)	58.0 (12.6)	54.3 (13.0)	57.4 (11.9)	65.4 (11.2)	<0.01	
Sex at birth						
Female	988 (38.8)	287 (40.0)	560 (41.3)	141 (29.9)	<0.01	
Male	1557 (61.2)	431 (60.0)	795 (58.7)	331 (70.1)		
Ethnicity						15
White	1893 (74.8)	514 (72.3)	992 (73.5)	387 (82.5)	<0.01	
South Asian	198 (7.8)	61 (8.6)	113 (8.4)	24 (5.1)		
Black	180 (7.1)	33 (4.6)	117 (8.7)	30 (6.4)		
Mixed/other	259 (10.2)	103 (14.5)	128 (9.5)	28 (6.0)		
Admission data						
Time from first symptom to admission (days)	8.0 (6.0–11.0)	9.0 (6.0–12.0)	8.0 (6.0–11.0)	8.0 (5.0–11.0)	0.03	294
Length of hospital stay (days)	8.0 (4.0–16.0)	6.0 (3.0–15.0)	8.0 (4.0–16.0)	9.0 (5.0–18.0)	<0.01	
PCR positive test	2164 (93.0)	597 (91.3)	1148 (93.3)	419 (94.8)	0.07	219
WHO Clinical Progression Scale					<0.01	77
Class 4 (no oxygen therapy)	392 (15.9)	136 (19.6)	182 (13.8)	74 (16.2)		
Class 5 (oxygen by mask or nasal prongs)	1055 (42.7)	300 (43.2)	562 (42.7)	193 (42.2)		
Class 6 (oxygen by non-invasive ventilation or high-flow nasal oxygen)	584 (23.7)	133 (19.2)	337 (25.6)	114 (24.9)		
Class 7–9 (admitted to ICU for intubation and mechanical ventilation)	437 (17.7)	125 (18.0)	236 (17.9)	76 (16.6)		
Complications during admission						
Proning required	469 (20.5)	138 (21.4)	253 (20.9)	78 (18.3)	0.42	261
Renal replacement therapy	104 (4.3)	21 (3.1)	57 (4.4)	26 (5.7)	0.08	99
Pulmonary embolism	242 (9.9)	83 (12.2)	121 (9.3)	38 (8.4)	0.06	108
Renal failure requiring haemodialysis	82 (3.4)	16 (2.3)	46 (3.5)	20 (4.4)	0.15	103
Antibiotics	1951 (78.6)	533 (75.8)	1041 (78.9)	377 (82.0)	0.04	63
Systemic steroids	1387 (57.3)	405 (59.0)	736 (57.1)	246 (55.0)	0.41	124
Anticoagulation	1104 (45.5)	319 (46.3)	576 (44.6)	209 (47.2)	0.57	121
Lowest eGFR (mL/min/1.73 m^2^)	81 (61–91)	88 (71–91)	80 (61–91)	65 (49–87)	<0.01	311
Alanine transaminase (U/L)	60 (34–109)	66 (36–117)	60 (34–108)	53 (30–98)	<0.01	244

Data are presented as mean (SD) or median (IQR) for continuous measures, and n (%) for categorical measures.

eGFR estimated glomerular filtration rate.

ICU, intensive care unit.

Compared with controls (n=718, aged 54.3±13.0 years), patients with cardiovascular risk factors (n=1355) and CVD (n=472) were on average 3 and 11 years older, respectively. Of those with CVD, 172/472 (36.4%) had ischaemic heart disease, 108/472 (22.9%) a prior history of myocardial infarction and 41/472 (8.7%) patients had a prior history of heart failure (online supplemental table 1). Patients with cardiovascular risk factors or CVD had more severe COVID-19 (WHO Clinical Progression Scale ≥6 430.5%, 41.5% vs 37.2%, p<0.01), higher rates of non-invasive ventilation (25.6%, 24.9%, vs 19.2%, p<0.01) and antibiotic use (78.9%, 82.0% vs 75.8%, p=0.04) but similar rates of systemic corticosteroid use (57.1%, 55.0% vs 59.0%, p=0.41) (table 1).

10.1136/openhrt-2024-002662.supp1 Supplementary data

### Full recovery from COVID-19 at 5 months and 12 months

**Figure 2:**
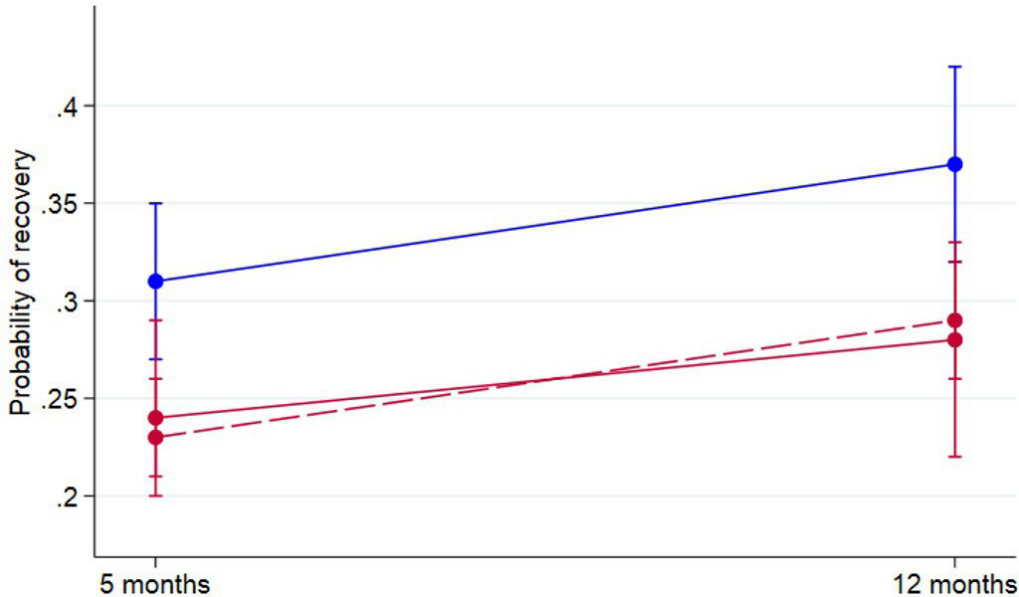
Recovery following COVID-19 stratified by disease/risk group. Probability of patient-perceived full recovery stratified by patients with established cardiovascular disease (red), patients at high risk of developing cardiovascular disease (dotted red), patients without either established cardiovascular disease or cardiovascular risk factors (blue).

Compared with controls, the proportion of patients with cardiovascular risk factors who reported full recovery at 5 months was lower (253/1098 (23%) vs 175/578 (30%), adjusted OR (aOR) 0.64, 95% CI 0.51 to 0.81). Similarly, the proportion of patients with CVD who reported full recovery at 5 months was lower (102/386 (26%), aOR 0.71, 95% CI 0.52 to 0.98). These reductions in full recovery persisted at 12 months in both those with cardiovascular risk factors (aOR 0.66, 95% CI 0.50 to 0.86) and established CVD (aOR 0.62, 95% CI 0.43 to 0.89) (table 2, figure 2). In an exploratory analysis, full recovery at 12 months remained significantly lower after adjustment for frailty in the patients with cardiovascular risk factors (aOR 0.74, 95% CI 0.56 to 0.99), but not in those with established CVD (aOR 0.96, 95% CI 0.64 to 1.43).

**Table 2:** Patient-reported recovery following hospitalisation with COVID-19 stratified by disease/risk group

	Control group n (%)	Disease/risk group n (%)	OR 95% CI	Adjusted OR* 95% CI
Established cardiovascular disease				
Full recovery at 5 months	174/578 (30)	102/386 (26)	0.84 (0.63 to 1.12)	0.71 (0.52 to 0.98)
Full recovery at 12 months	152/416 (36)	84/285 (29)	0.71 (0.51 to 0.98)	0.62 (0.43 to 0.89)
High risk of developing cardiovascular disease				
Full recovery at 5 months	174/578 (30)	253/1098 (23)	0.68 (0.54 to 0.85)	0.64 (0.50 to 0.81)
Full recovery at 12 months	152/416 (36)	242/841 (29)	0.70 (0.54 to 0.89)	0.66 (0.50 to 0.86)

*Adjusted for age, sex, ethnicity and COVID-19 severity.

## Physical, physiological and cognitive recovery

**Figure 3:**
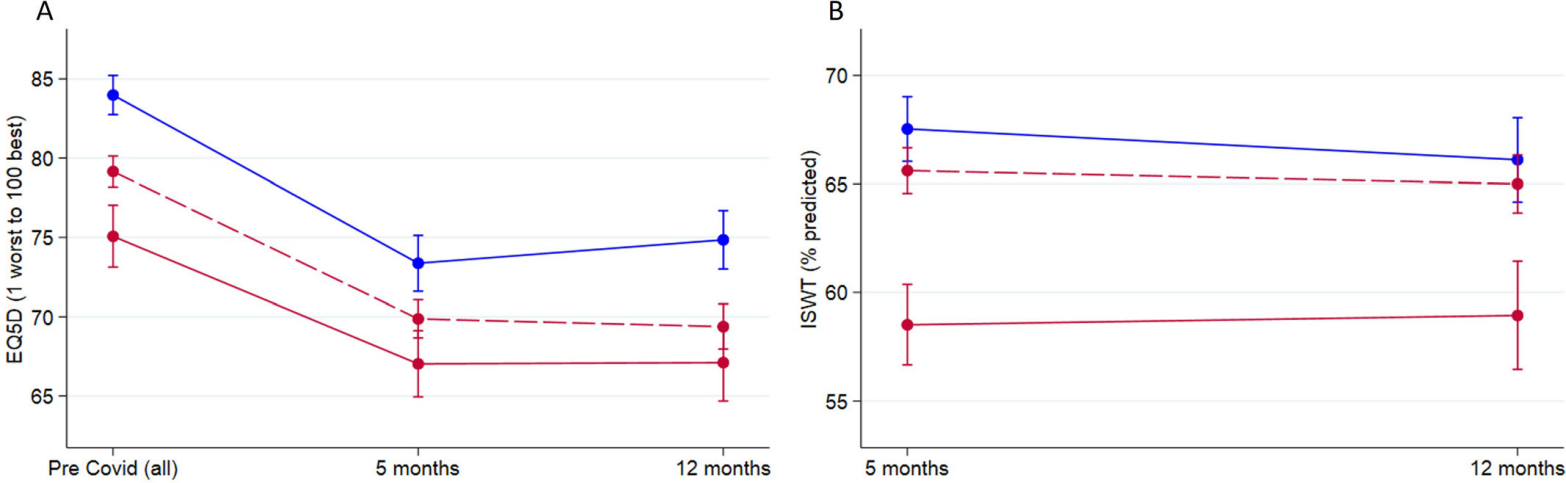
Patient-reported outcome measures at 5 and 12 months stratified by disease/risk group. Patient-related outcome measures of quality of life (EQ-5D-5L) prior to COVID-19 infection and at 5 and 12 months stratified by disease group (A). Physiological assessment (ISWT) of maximal predicted walking distance adjusted for age at 5 and 12 months (B). Patients with established cardiovascular disease (red), patients at high risk of developing cardiovascular disease (dotted red), patients without either established cardiovascular disease or cardiovascular risk factors (blue). EQ-5D-5L, EuroQol 5D Questionnaire; ISWT, incremental shuttle walk test.

Patient-reported outcome measures, metrics of physiological recovery, cognitive recovery, frailty and biomarkers are shown in tables 3 and 4 stratified by the three groups. At 5 months, as expected, patients with CVD had higher rates of inactivity compared with controls (56% vs 43%, p<0.001). In keeping with this, patients with CVD had higher frailty scores (3.10 vs 2.46, p<0.001) with higher proportions of patients exhibiting symptoms of increased anxiety (GAD7 score >8 33% vs 19%, p=<0.001) and low mood (PHQ-9 score ≥10 39% vs 22%, p<0.001) (table 3). In contrast at 5 months, patients with cardiovascular risk factors had comparable rates of inactivity to controls (44% vs 43%, p=0.748) and less marked elevations in frailty scores (2.78 vs 2.46, p<0.001) with smaller differences in the proportions of patients exhibiting symptoms of increased anxiety (GAD-7 score >8 25% vs 19%, p=0.003) and low mood (PHQ-9 score ≥10 30% vs 22%, p=0.001). At 12 months, the control group was more physically active with inactivity rates reducing from 43% to 36%, however, there were no differences in the rates of inactivity in patients with CVD (56%; 5 months vs 57%; 12 months) or patients with cardiovascular risk factors (44%; 5 months vs 47%; 12 months) (tables 3 and 4). Between 5 and 12 months, there were no differences in the maximum incremental shuttle walk predicted distances in the controls (68% vs 66%), CVD (59% vs 59%) and cardiovascular risk factors (66% vs 65%) (tables 3 and 4, figure 3).

**Table 3:** Recovery measures at 5 months stratified by disease/risk group

	Control (n=718)	High risk of developing CVD (n=1355)	Established CVD (n=472)	Missing values
Recovered from COVID-19				483
Yes	31% (27 to 35)	23% (21 to 26)	24% (20 to 28)	
Unsure	20% (16 to 23)	19% (17 to 22)	21% (17 to 25)	
No	49% (45 to 53)	57% (54 to 60)	55% (50 to 60)	
Patient-perceived recovery				
Symptom number	8 (8 to 9)	10 (9 to 10)	11 (10 to 12)	431
Dyspnoea				
Dyspnoea-12 score (1–36)	5 (5 to 6)	6 (6 to 7)	8 (7 to 9)	261
Breathlessness before (1–10)	0.78 (0.59 to 0.97)	1.15 (1.03 to 1.27)	1.93 (1.66 to 2.21)	987
Breathlessness now	2.32 (2.12 to 2.53)	2.88 (2.69 to 3.06)	3.04 (2.71 to 3.36)	620
Breathlessness change	1.59 (1.30 to 1.88)	1.81 (1.62 to 2.00)	1.21 (0.88 to 1.54)	1117
Fatigue				
FACIT-Fatigue (0–52)	37 (36 to 38)	34 (34 to 35)	32 (31 to 34)	262
FACIT-Fatigue before (1–10)	1.12 (0.90 to 1.36)	1.47 (1.34 to 1.60)	2.08 (1.77 to 2.39)	998
FACIT-Fatigue now	3.18 (3.08 to 3.46)	3.72 (3.53 to 3.92)	3.96 (3.62 to 4.30)	627
FACIT-Fatigue change	2.00 (1.80 to 2.32)	2.21 (1.97 to 2.44)	1.85 (1.44 to 2.25)	1123
EQ-5D-5L				
EQ-5D-5L before (1–100)	83.98 (82.74 to 85.22)	79.15 (78.17 to 80.13)	75.07 (73.12 to 77.03)	470
EQ-5D-5L now	73.37 (71.67 to 75.13)	69.87 (68.67 to 71.07)	67.03 (64.94 to 69.12)	449
EQ-5D-5L change	−11.61 (−13.48 to −10.86)	−9.75 (−10.98 to −8.51)	−8.10 (−10.18 to −6.02)	855
General Practice Physical Activity Questionnaire				260
Active	19% (16 to 22)	18% (16 to 20)	16% (12 to 19)	
Moderately active	22% (19 to 26)	20% (18 to 22)	15% (12 to 19)	
Moderately inactive	14% (11 to 17)	17% (15 to 19)	11% (8 to 15)	
Inactive	43% (39 to 47)	44% (41 to 47)	56% (52 to 61)	
Physiological recovery				
ISWT distance (m)	496 (472 to 521)	384 (371 to 396)	316 (294 to 338)	923
ISWT % predicted	68 (66 to 69)	66 (65 to 67)	59 (57 to 60)	927
Handgrip strength (kg)	29.9 (29.2 to 30.6)	28.8 (28.2 to 29.3)	27.2 (26.3 to 28.1)	370
Short Physical Performance Battery ≤10	42% (38 to 46)	53% (50 to 56)	61% (56 to 66)	463
Cognitive recovery				
Anxiety: GAD-7>8	19% (16 to 22)	25% (22 to 27)	33% (28 to 37)	241
Depression: PHQ-9≥10	22% (19 to 26)	30% (27 to 32)	39% (34 to 44)	247
Montreal Cognitive Assessment ≤23	14% (11 to 17)	15% (12 to 17)	18% (14 to 22)	492
Frailty				
Rockwood Frailty Score	2.46 (2.36 to 2.56)	2.78 (2.72 to 2.83)	3.10 (2.99 to 3.21)	269
Rockwood Frailty level				269
Very fit	20% (17 to 23)	10% (8 to 11)	8% (5 to 1)	
Well or managing well	64% (60 to 67)	68% (66 to 71)	60% (55 to 65)	
Vulnerable	11% (9 to 14)	16% (14 to 18)	20% (16 to 24)	
Mildly frail	2% (1 to 4)	3% (2 to 4)	6% (3 to 8)	
Moderate or higher frail severity	1% (0 to 2)	2% (1 to 3)	5% (3 to 7)	
Nottingham ADL scale (1–22)	19 (18 to 19)	18 (18 to 18)	17 (16 to 17)	
Serum biomarkers				
CRP (mg/L; median)	3.3 (2.8 to 3.8)	4.1 (3.9 to 4.1)	4.1 (3.5 to 4.6)	391
CRP>5 mg/L	19% (16 to 22)	26% (24 to 29)	25% (21 to 30)	
HbA1c (mmol/mL; median)	37.9 (37.4 to 38.3)	40.7 (40.1 to 41.4)	41.0 (20.1 to 41.9)	830
HbA1c				
<42 mmol/mL	80% (76 to 84)	54% (0.51 to 0.57)	53% (48 to 59)	
42–47 mmol/mL	16% (12 to 19)	18% (0.16 to 0.21)	19% (15 to 24)	
>47 mmol/mL	4% (2 to 6)	27% (0.25 to 0.30)	27% (22 to 32)	
BNP≥100 or NT-proNP≥400	3% (1 to 4)	2% (0.01 to 0.03)	16% (11 to 21)	876

ADL, Activity of Daily Living; BNP, brain natriuretic peptide; CRP, C reactive protein; EQ-5D-5L, EuroQol 5D-5L Questionnaire; FACIT-Fatigue, Functional Assessment of Chronic Illness Therapy-fatigue scale; GAD-7, Generalised Anxiety Disorder assessment; ISWT, incremental shuttle walk test; NT-proBNP, N-terminal pro hormone brain natriuretic peptide; PHQ-9, Patient Health Questionnaire depression test.

**Table 4:** Recovery measures at 12 months stratified by disease/risk group

	Control (n=492)	High risk of developing CVD (n=984)	Established CVD (n=351)	Missing values
Recovered from COVID-19				304
Yes	37% (32 to 42)	29% (26 to 32)	28% (22 to 33)	
Unsure	21% (17 to 25)	21% (18 to 24)	22% (17 to 28)	
No	42% (37 to 47)	50% (46 to 53)	50% (44 to 56)	
Patient-prceived recovery				
Symptom number	9 (8 to 10)	10 (10 to 11)	12 (11 to 13)	284
Dyspnoea				
Dyspnoea-12 score	4 (4 to 5)	6 (5 to 6)	8 (7 to 9)	288
Breathlessness before (1–10)	0.80 (0.58 to 1.02)	1.10 (0.93 to 1.26)	1.90 (1.58 to 2.22)	712
Breathlessness now	1.85 (1.58 to 2.11)	2.33 (2.14 to 2.52)	2.88 (2.55 to 3.21)	380
Breathlessness change	1.16 (0.83 to 1.49)	1.44 (1.20 to 1.68)	0.87 (0.37 to 1.36)	916
Fatigue				
FACIT-Fatigue	39 (37 to 40)	35 (35 to 36)	34 (33 to 35)	288
FACIT-Fatigue before (1–10)	1.22 (0.94 to 1.50)	1.46 (1.30 to 1.63)	2.02 (1.70 to 2.34)	721
FACIT-Fatigue now	2.68 (2.39 to 2.98)	3.22 (3.03 to 3.41)	3.85 (3.45 to 4.26)	372
FACIT-Fatigue change	1.37 (0.92 to 1.81)	1.83 (1.52 to 2.15)	2.02 (1.51 to 2.53)	918
EQ-5D-5L				
EQ-5D-5L before (1–100)	84.04 (82.63 to 85.45)	79.14 (77.85 to 80.42)	74.47 (72.43 to 76.52)	289
EQ-5D-5L now	74.85 (73.00 to 76.69)	69.38 (67.96 to 70.81)	66.11 (64.68 to 69.53)	340
EQ-5D-5L change	−9.78 (−12.18 to −7.39)	−10.67 (−12.06 to −9.27)	−8.00 (−10.75 to −5.25)	581
General Practice Physical Activity Questionnaire				316
Active	24% (20 to 29)	16% (14 to 19)	14% (10 to 18)	
Moderately active	21% (17 to 25)	21% (18 to 24)	16% (11 to 20)	
Moderately inactive	17% (13 to 20)	15% (12 to 17)	12% (8 to 16)	
Inactive	36% (31 to 41)	47% (44 to 51)	57% (51 to 63)	
Physiological recovery				
ISWT distance (m)	537 (508 to 566)	419 (400 to 438)	363 (333 to 393)	754
ISWT % predicted	66 (64 to 68)	65 (64 to 66)	59 (56 to 61)	750
Handgrip strength (kg)	30.2 (29.4 to 31.1)	29.8 (29.1 to 30.4)	27.5 (26.3 to 28.7)	258
Short Physical Performance Battery ≤10	37% (32 to 42)	49% (45 to 52)	61% (54 to 68)	511
Cognitive recovery				
Anxiety: GAD-7>8	19% (15 to 23)	23% (20 to 26)	30% (25 to 36)	271
Depression: PHQ-9≥10	20% (16 to 24)	25% (22 to 28)	33% (27 to 39)	988
Montreal Cognitive Assessment ≤23	5% (3 to 7)	9% (7 to 11)	10% (7 to 13)	492
Frailty				
Rockwood Frailty Score	2.34 (2.24 to 2.43)	2.73 (2.66 to 2.79)	3.17 (3.05 to 3.30)	195
Rockwood Frailty level				
Very fit	25% (21 to 29)	12% (9 to 14)	6% (3 to 8)	
Well or managing well	60% (55 to 64)	69% (66 to 72)	60% (54 to 66)	
Vulnerable	11% (8 to 14)	14% (12 to 17)	22% (17 to 27)	
Mildly frail	2% (1 to 4)	3% (2 to 4)	7% (4 to 10)	
Moderate or higher frail severity	0% (0 to 1)	2% (1 to 3)	4% (2 to 7)	
Nottingham ADL scale (1–22 (best))	19 (19 to 20)	18 (18 to 19)	18 (17 to 18)	312
Serum biomarkers				
CRP (mg/L; median)	3.5 (2.9 to 4.2)	4.0 (3.8 to 4.2)	4.2 (3.7 to 4.6)	385
CRP>5 mg/L	20% (16 to 24)	24% (21 to 27)	30% (25 to 36)	
HbA1c (mmol/mL; median)	37.8 (37.2 to 38.3)	40.8 (40.2 to 41.4)	41.3 (39.4 to 43.1)	720
HbA1c				
<42 mmol/mL	83% (79 to 88)	54% (50 to 58)	53% (46 to 59)	
42–47 mmol/mL	11% (7 to 15)	17% (14 to 21)	19% (14 to 25)	
>47 mmol/mL	6% (3 to 8)	28% (24 to 31)	27% (21 to 33)	
BNP≥100 or NT-proNP≥400	3% (1 to 5)	2% (1 to 4)	17% (11 to 24)	929

ADL, Activity of Daily Living; BNP, brain natriuretic peptide; CRP, C reactive protein; EQ-5D, EuroQol 5D Questionnaire; FACIT-Fatigue, Functional Assessment of Chronic Illness Therapy-fatigue scale; GAD-7, Generalised Anxiety Disorder assessment; ISWT, incremental shuttle walk test; NT-proBNP, N-terminal pro hormone brain natriuretic peptide; PHQ-9, Patient Health Questionnaire depression test.

## Discussion

In this prospective observational study of hospitalised COVID-19 survivors in the UK fewer than one in three patients with cardiovascular comorbidities or at high risk of developing CVD had complete patient perceived physical, mental and cognitive recovery up to 1 year following SARS-CoV-2 infection. The profound functional deterioration and lower quality of life that have been previously reported at 5 months persist at 1 year, particularly for those with established CVD or with cardiovascular risk factors. Compared with controls who were younger and more physically active, patients at high risk of developing CVD had a similar recovery profile to those with established CVD. We have demonstrated that the presence of either pre-COVID-19 CVD or cardiovascular risk factors are associated with a greater than 30% relative risk reduction in the probability of complete patient-perceived recovery at 1 year following adjustment for age, sex, ethnicity and the severity of hospitalised COVID-19 illness. This observation may herald the evaluation of early targeted interventions for individuals at increased cardiovascular risk to support their recovery following SARS-CoV-2 infection.

Trajectories following hospitalisation with respiratory infections are comparable to patients admitted with heart failure, namely, there is a high short-term risk of complications during the early post-drome recovery period which extends to 6 months after discharge.^11^ In the early post-drome period, the interaction between respiratory infections and cardiovascular risk has been well documented to the extent that specific viruses such as influenza are temporally associated with short-term cardiovascular complications.^12–14^ Shared chronic disease risk factors implicated in both severe infections and CVD may account for an accelerated progression of CVD rather than the pathogen itself. Preventative strategies that improve vaccination uptake against circulating strains of viral pathogens, as exemplified in the influenza vaccination after myocardial infarction trial, have been underappreciated as a tool to attenuate the risk of cardiovascular complications.^15^ Vaccination programmes continue to have relevance in the context of long COVID in light of the strong association between COVID-19 vaccination and the reduced population risk of long COVID.^16^ However, whether COVID-19 vaccination reduces cardiovascular complications in a similar manner to influenza vaccination remains unproven.

We, and others, have shown that the risk of myocardial involvement after hospitalisation for COVID-19 in those with elevated cardiac troponin during the hospital admission is low.^17–19^ As the recovery profiles presented in our study are related to pre-COVID-19 cardiovascular status, we posit that it is deconditioning following the hospital admission which limits the rate of recovery, as has been noted in different frailty phenotypes.^20^ Sarcopenic measures in our study, such as the incremental shuttle walk distance, were 18%–31% lower in cardiovascular groups compared with the reference group after adjustment for age, sex and ethnicity: this equates to an average walking speed of less than 1 m/s. By comparison, the walking distances achieved at 1 year in the cardiovascular groups are comparable with age and sex-matched reference distances for patients who are routinely referred to outpatient cardiac rehabilitation programmes, namely 66% of the distance achieved by a healthy population.^21^ Despite access to online peer-support communities which included exercise tutorials as part of the ‘Your COVID Recovery’ we detected only minimal increases in the walking distance over 1 year which did not reach the threshold for a meaningful clinical improvement.^22^ Targeted interventions to address deconditioning in patients with established CVD and cardiovascular risk factors may help alleviate symptoms in those with delayed recovery following SARS-CoV-2 infection. In this regard, metformin prescribed early in the course of an acute symptomatic SARS-CoV-2 infection has been shown to reduce the risk of long COVID at 180 days.^23 24^ Further validation of metabolic modulation to improve the symptom burden of long COVID is required.^25^

There are some limitations to acknowledge in our study. The definition of recovery following SARS-CoV-2 infection was a subjective assessment based on patient-reported outcome measures. These measures may reflect additional complex social and economic factors as well as a patient’s intrinsic motivation which were not formally reported in the data set. Despite rates of missingness >10% for several variables, this cohort represents the largest prospective observational sample of patients with confirmed SARS-CoV-2 infection and detailed cardiovascular grouping including a control group cohort at low cardiovascular risk. A ‘healthy dropout’ effect may be present which limits the use of multiple imputations in our modelling as missing data was unlikely to be missing at random. Furthermore, the absence of a negative control without COVID-19 illness or un-hospitalised COVID-19 illness limits any assessment of causality and outcomes for milder disease. Importantly, differences in covariates between groups may account for some of the observations and causality cannot be inferred from this study design. Interventions established during the course of the COVID-19 pandemic that reduce the severity of COVID-19 illness (ie, vaccination) may reduce the significance of our findings.

### Conclusion

Patients with either CVD or at high risk of developing CVD had a lower probability of complete patient-perceived physical, physiological and cognitive recovery up to 1 year following hospitalisation with SARS-CoV-2 infection. Targeted interventions for patients with CVD or risk factors may reduce the health and socioeconomic burden of patients with long COVID.

## Data Availability

Data are available upon reasonable request.

## References

[1] Linschoten M , Uijl A , Schut A , et al . The CAPACITY-COVID collaborative consortium and LEOSS study group. clinical presentation, disease course, and outcome of COVID-19 in hospitalized patients with and without pre-existing cardiac disease: a cohort study across 18 countries. Eur Heart J 2022;43:1104–20. 10.1093/eurheartj/ehab656 34734634

[2] Ballering AV , van Zon SKR , Olde Hartman TC , et al . Persistence of somatic symptoms after COVID-19 in the Netherlands: an observational cohort study. Lancet 2022;400:452–61. 10.1016/S0140-6736(22)01214-4 35934007 PMC9352274

[3] Houchen-Wolloff L , Poinasamy K , Holmes K , et al . Joint patient and clinical priority setting to identify 10 key research questions regarding the long-term sequelae of COVID-19. Thorax 2022;77:717–20. 10.1136/thoraxjnl-2021-218582 35354642 PMC9209667

[4] Brightling CE , Evans RA . Long COVID: which symptoms can be attributed to SARS-Cov-2 infection. Lancet 2022;400:411–3. 10.1016/S0140-6736(22)01385-X 35933996 PMC9352303

[5] Evans RA , McAuley H , Harrison EM , et al . Physical, cognitive, and mental health impacts of COVID-19 after hospitalisation (PHOSP-COVID): a UK multicentre, prospective cohort study. Lancet Respir Med 2021;9:1275–87. 10.1016/S2213-2600(21)00383-0 34627560 PMC8497028

[6] Reese JT , Blau H , Casiraghi E , et al . Generalisable long COVID subtypes: findings from the NIH N3C and RECOVER programmes. EBioMedicine 2023;87:104413. 10.1016/j.ebiom.2022.104413 36563487 PMC9769411

[7] Hastie CE , Lowe DJ , McAuley A , et al . Outcomes among confirmed cases and a matched comparison group in the long-COVID in Scotland study. Nat Commun 2022;13:5663. 10.1038/s41467-022-33415-5 36224173 PMC9556711

[8] Elneima O , McAuley HJC , Leavy OC , et al . Cohort profile: post-Hospitalisation COVID-19 (PHOSP-COVID) study. Int J Epidemiol 2023. 10.1093/ije/dyad165 PMC10859139 39429158

[9] WHO working group on the clinical characterisation and management of COVID-19 infection. A minimal common outcome measure set for COVID-19 clinical research. Lancet Infect Dis 2020;20:e192–7. 32539990 10.1016/S1473-3099(20)30483-7 PMC7292605

[10] White IR , Carlin JB . Bias and efficiency of multiple imputation compared with complete-case analysis for missing Covariate values. Stat Med 2010;29:2920–31. 10.1002/sim.3944 20842622

[11] Dharmarajan K , Hsieh AF , Kulkarni VT , et al . Trajectories of risk after hospitalization for heart failure, acute myocardial infarction, or pneumonia: restrospective cohort study. BMJ 2015;350:h411. 10.1136/bmj.h411 25656852 PMC4353309

[12] Smeeth L , Thomas SL , Hall AJ , et al . Risk of myocardial infarction and stroke after acute infection or vaccination. N Engl J Med 2004;351:2611–8. 10.1056/NEJMoa041747 15602021

[13] Sipilä PN , Lindbohm JV , Batty GD , et al . Severe infection and risk of cardiovascular disease: a multicohort study. Circulation 2023;147:1582–93. 10.1161/CIRCULATIONAHA.122.061183 36971007

[14] Nguyen JL , Yang W , Ito K , et al . Seasonal influenza infections and cardiovascular mortality. JAMA Cardiol 2016;1:274–81. 10.1001/jamacardio.2016.0433 27438105 PMC5158013

[15] Fröbert O , Götberg M , Erlinge D , et al . Influenza vaccination after myocardial infarction: a randomized, double-blind, placebo-controlled, multicenter trial. Circulation 2021;144:1476–84. 10.1161/CIRCULATIONAHA.121.057042 34459211

[16] Lundberg-Morris L , Leach S , Xu Y , et al . Covid-19 vaccine effectiveness against post-Covid-10 condition among 589,772 individuals in Sweden: population based cohort study. BMJ 2023;383:e076990. 10.1136/bmj-2023-076990 37993131 PMC10666099

[17] Artico J , Shiwani H , Moon JC , et al . Myocardial involvement after hospitalization for COVID-19 complicated by troponin elevation: a prospective, multicenter, observational study. Circulation 2023;147:364–74. 10.1161/CIRCULATIONAHA.122.060632 36705028 PMC9889203

[18] Raman B , McCracken C , Cassar MP , et al . Multiorgan MRI findings after hospitalisation with COVID-19 in the UK (C-MORE): a prospective, multicentre, observational cohort study. Lancet Respir Med 2023;11:1003–19. 10.1016/S2213-2600(23)00262-X 37748493 PMC7615263

[19] Singh T , Kite TA , Joshi SS , et al . MRI and CT coronary angiography in survivors of COVID-19. Heart 2022;108:46–53. 10.1136/heartjnl-2021-319926 34615668 PMC8503921

[20] McAuley HJC , Evans RA , Bolton CE , et al . Prevalence of physical frailty, including risk factors, up to 1 year after hospitalisation for COVID-19 in the UK: a multicentre, longitudinal cohort study. EClinicalMedicine 2023;57:101896. 10.1016/j.eclinm.2023.101896 36936404 PMC10005893

[21] Alotaibi JFM , Doherty P . Evaluation of determinants of walking fitness in patients attending cardiac rehabilitation. BMJ Open Sport Exerc Med 2016;2:e000203. 10.1136/bmjsem-2016-000203 PMC5569262 28879036

[22] Singh SJ , Jones PW , Evans R , et al . Minimum clinical important improvement for the incremental shuttle walking test. Thorax 2008;63:775–7. 10.1136/thx.2007.081208 18390634

[23] Bramante CT , Huling JD , Tignanelli CJ , et al . Randomized trial of metformin, Ivermectin, and fluvoxamine for COVID-19. N Engl J Med 2022;387:599–610. 10.1056/NEJMoa2201662 36070710 PMC9945922

[24] Bramante CT , Beckman KB , Mehta T , et al . Metformin reduces SARS-Cov-2 in a phase 3 randomized placebo controlled clinical trial. medRxiv 2023. 10.1101/2023.06.06.23290989

[25] Finnigan LEM , Cassar MP , Koziel MJ , et al . Efficacy and tolerability of an endogenous metabolic modulator (Axa1125) in fatigue-predominant long COVID: a single-centre, double-blind, randomised controlled phase 2A pilot study. EClinicalMedicine 2023;59:101946. 10.1016/j.eclinm.2023.101946 37223439 PMC10102537

